# Structural identification of conserved RNA binding sites in herpesvirus ORF57 homologs: implications for PAN RNA recognition

**DOI:** 10.1093/nar/gky1181

**Published:** 2018-11-20

**Authors:** Richard B Tunnicliffe, Colin Levy, Hilda D Ruiz Nivia, Rozanne M Sandri-Goldin, Alexander P Golovanov

**Affiliations:** 1 Manchester Institute of Biotechnology, School of Chemistry, Faculty of Science and Engineering, The University of Manchester, Manchester M1 7DN, UK; 2 Biomolecular Analysis Core Facility, Faculty of Biology, Medicine and Health, University of Manchester, Manchester M13 9PT, UK; 3 Department of Microbiology and Molecular Genetics, School of Medicine, University of California, Irvine, CA 92697-025, USA

## Abstract

Kaposi's sarcoma-associated herpesvirus (KSHV) transcribes a long noncoding polyadenylated nuclear (PAN) RNA, which promotes the latent to lytic transition by repressing host genes involved in antiviral responses as well as viral proteins that support the latent state. KSHV also expresses several early proteins including ORF57 (Mta), a member of the conserved multifunctional ICP27 protein family, which is essential for productive replication. ORF57/Mta interacts with PAN RNA via a region termed the Mta responsive element (MRE), stabilizing the transcript and supporting nuclear accumulation. Here, using a close homolog of KSHV ORF57 from herpesvirus saimiri (HVS), we determined the crystal structure of the globular domain in complex with a PAN RNA MRE, revealing a uracil specific binding site that is also conserved in KSHV. Using solution NMR, RNA binding was also mapped within the disordered N-terminal domain of KSHV ORF57, and showed specificity for an RNA fragment containing a GAAGRG motif previously known to bind a homologous region in HVS ORF57. Together these data located novel differential RNA recognition sites within neighboring domains of herpesvirus ORF57 homologs, and revealed high-resolution details of their interactions with PAN RNA, thus providing insight into interactions crucial to viral function.

## INTRODUCTION

Kaposi's sarcoma herpesvirus (KSHV) is a γ-herpesvirus that can cause malignancies including Kaposi's sarcoma (KS), Primary Effusion Lymphoma and Multicentric Castleman's Disease, primarily in immunocompromised individuals such as AIDS patients and transplant recipients (1). In common with all herpesviruses, KSHV has a tightly regulated biphasic life cycle comprised of either a quiescent latent or a lytic state. In the latent state, the viral genome is maintained as a circular episome within the host cell nucleus with highly restricted viral protein expression in order to maintain the genome in the dividing cells. Upon transition to the lytic state, the expression of the remaining viral genes occurs in a temporally regulated cascade: immediate early (IE), early (E) and late (L), with the host cell machinery being hijacked to produce viral progeny. For the initiation of the lytic cycle, the KSHV transcription factor ORF50/Rta is necessary and sufficient to activate the gene expression cascade that has a profound influence on the composition of the host cell nucleus (2–4). It has been demonstrated that ORF50 promotes the expression of several viral transcripts, two prominent examples of which are a long non-coding polyadenylated nuclear (PAN) RNA (also called nut1, T1.1) (5,6) and the ORF57/Mta protein (here called ksORF57, to discriminate it from homologous ORF57 proteins found in other herpesviruses) (7–10). PAN RNA accumulates in the nucleus becoming the most abundant transcript, and suppresses the expression of host genes involved in antiviral responses (11–14). This accumulation is supported by a 79-nucleotide sequence termed the expression and nuclear retention element (ENE), which interacts with the poly(A) tail forming a triple-helix structure that provides resistance to cellular RNA degradation machinery (15–18). As part of its function, PAN RNA interacts with cellular transcription factors such as IRF4 (14), and chromatin modifiers such as histone H3K27 demethylases UTX and JMJD3, and the lysine methyltransferase MLL2 (19). Several PAN RNA interaction sites have been identified in the KSHV episome and the cellular genome, indicating that this viral long non-coding RNA modulates viral and cellular gene expression (20). ksORF57 is also a potent multifunctional regulator of gene expression that functions at a post-transcriptional level (21,22), and is necessary for productive viral replication (23,24). ksORF57 binds selectively to transcripts, especially intronless viral RNAs, stabilizing them, which results in their nuclear accumulation (25–29). It has been demonstrated that ksORF57 can promote mRNA export via recruiting components of the hTREX complex and also enhance the translation of some intronless viral mRNAs (26,30–33). Significantly, PAN RNA is one of the binding partners of ksORF57, and this interaction is necessary for its nuclear accumulation and is essential for the subsequent replication of the virus (23,24,28,29,34).

As part of its RNA stabilization and nuclear export functions, ksORF57 interacts with other viral proteins including ORF50 (35) and also numerous cellular proteins with functions in RNA splicing and export such as SR-proteins, ALYREF, UIF, PABP and hnRNPK (31,32,36–39). ALYREF is able to interact with PAN RNA in concert with ksORF57, while the interaction of PABPC1 with ksORF57 also leads to enhancing PAN stability (27,37,40). The RNA binding sites within the ksORF57 protein sequence have been inferred from sequence comparisons and tested by monitoring the interactions of mutated proteins. Three nuclear localization sequences (NLSs) within the predicted intrinsically disordered N-terminal domain (an arginine-rich region) have been shown to contribute to RNA interactions (26). Also mutation of an isolated RGG sequence (aa372–374) prevented RNA interaction (41). Homologs of ksORF57 have been identified in the genomes of all herpesviruses currently sequenced, and the region of sequence conservation is a C-terminal domain of approximately 270 residues (42). The structure of the homologous C-terminal domain from HSV-1 ICP27 showed that this region, termed the ICP27-homology domain (IHD), adopts a novel protein fold (43,44). The ICP27 IHD structure revealed a stable homo-dimer, a feature also detected in ksORF57 and other homologs in α- and γ-herpesviruses (41,42,45,46), while the β-herpesvirus homolog UL69 from HCMV forms a tetramer (47,48). Recently, both monomeric and dimeric crystal structures of the C-terminal domain of ksORF57 were solved at 3.1 and 3.5 Å resolution, respectively (49). Proteins from within the same α-, β- or γ-subfamily share closer sequence homology to each other than between subfamilies. For example, the α-herpesvirus ICP27 C-terminal domain is only 16% identical to ksORF57, whereas the γ-herpesvirus homolog (also called ORF57) from Herpesvirus saimiri (HVS), here called hvsORF57, shares 30% sequence identity with ksORF57 (Supplementary Figure S1). The C-terminal domain structure of ICP27 showed that it contains an extensive positively charged arginine patch on one face of the protein (43,44). While this has not been tested, this feature could potentially mediate interactions with nucleotides.

The sequence and structural features of RNAs that bind to ksORF57 have been studied; although a universal binding motif was not revealed, several contributory factors were identified. These include AT-content (22), and specific regions of transcripts termed Mta-responsive elements (MREs), such as those identified within PAN RNA (27,29,50), which are transferrable, and a motif within the KSHV ORF59 transcript (51,52). The MRE of PAN RNA was initially predicted to form a stem–loop structure, and mutations in the loop weakened ORF57 interactions, however, more recent structural data of the full PAN RNA sequence indicated a more complex architecture, although the ORF57 interaction site remained within a non-base paired loop (27,50,53). Several other ksORF57 binding sites distributed throughout the PAN RNA sequence have also been identified (53). Further clues to ksORF57 RNA specificity can be inferred from studies of γ-herpesvirus homologs, which have indicated that codon bias can have a considerable effect on ORF57 mediated processes (54,55). Uniquely, a short GAAGRG motif was identified to bind specifically to an intrinsically disordered region from the N-terminal domain of Herpesvirus saimiri ORF57 (hvsORF57) (56,57). Overall, the determinants for ksORF57 binding to RNA remain unclear, and current studies are suggestive of possible binding sites throughout the protein's structured C-terminal and unstructured N-terminal domains, and each site has unresolved nucleotide sequence specificities.

Due to the functional importance of the RNA interactions with ORF57 homologues, particularly with PAN RNA in KSHV, here we have explored the nucleotide sequence specificity, protein structure and RNA binding sites by NMR and X-ray crystallography. We used NMR to explore the RNA binding properties and map binding sites within the ksORF57 N-terminal domain, revealing specificity similar to that of an equivalent region of hvsORF57. We used surface plasmon resonance to explore the similarities and differences in binding of a PAN RNA MRE oligonucleotide fragment to IHDs of ICP27, UL69, hvsORF57 and ksORF57. We also obtained the high-resolution structure of the C-terminal IHD from hvsORF57 in both the presence and absence of a PAN RNA MRE oligonucleotide fragment. The data revealed a RNA binding site at the homo-dimer interface conserved in ksORF57 and other γ-herpesviruses. Together the data provides detailed high-resolution structure of hvsORF57, the closest ksORF57 homologue, revealing the molecular origins of the specificity for PAN RNA. Comparison with the archetype ICP27 structure indicated differences that provided an explanation for the lack of MRE recognition by the homologous protein ICP27 from the α-herpesvirus subfamily. The site-specific characterizations of RNA binding sites will facilitate more targeted functional studies in the future to unravel the importance of these discovered sites for KSHV replication.

## MATERIALS AND METHODS

### Cloning and expression

DNA encoding an HRV3C protease cleavable N-terminal thioredoxin tag and HVS ORF57 residues 146–417 (hvsORF57Δ146) and KSHV ORF57 residues 153–455 (ksORF57Δ153) were obtained by gene synthesis (Invitrogen) in codon optimized form for expression in *E. coli*. DNA fragments were each cloned into the NdeI and XhoI restriction sites of pET-21a(+) (Merek). Protein was expressed in *Escherichia coli* strain T7 express LysY (New England Biolabs). Terrific Broth (Sigma) supplemented with 50 μg/ml ampicillin and 0.1 mM ZnSO_4_ was inoculated with 1% (v/v) overnight pre-culture. Culture density was monitored at 600 nm until an absorbance of 0.6, at which point protein expression was induced with 0.25 mM IPTG and incubation continued for 18 h at 20°C. Cells were pelleted by centrifugation (5000 g, 20 m). Selenomethionine (SeMet) labelled protein was obtained by growing cells in M9 minimal media in place of terrific broth, using the protocol described by Van Duyne *et al.* (58).

### Protein purification

Purification of ksORF57^68–178^ was carried out as previously described by His-tag purification followed by size-exclusion chromatography (39). HCMV UL69^200–540^ and HSV-1 ICP27Δ241 were purified via Strep-affinity tag and size-exclusion chromatography as previously described (43,48). ksORF57Δ153 and hvsORF57Δ146 were purified by the same method; cell pellets were resuspended in ice cold running buffer (50 mM HEPES, 500 mM NaCl, 50 mM l-Arg, 50 mM l-Glu, 1 mM TCEP, pH 7.9) supplemented with 0.5% (v/v) Triton X-100, DNase, RNase and EDTA free protease inhibitor (Roche). l-Arg·l-Glu was added to improve protein sample solubility and stability (59). The cell suspension was lysed by sonication and clarified by centrifugation (35 000 g, 30 m, 4°C) then the supernatant was passed through a 0.2 μm filter. The supernatant was purified on an AKTA prime with a 5 ml StrepTrap HP column, and bound material was eluted with 5 mM d-desthiobiotin in running buffer. The thioredoxin tag was cleaved by incubation with HRV3C protease for 16 h at 4°C. Finally the protein was purified on a Superdex 75 26/600 column (GE healthcare) pre-equilibrated in gel filtration buffer (20 mM HEPES, 150 mM NaCl, 50 mM l-Arg, 50 mM l-Glu, 1 mM TCEP, pH 7.4). Purified ORF57 constructs were concentrated in Vivaspin 500 centrifugal devices with a 5 kDa MWCO (Sartorius Stedim Biotech GmbH) as necessary prior to further experiments.

### Crystallization

For free hvsORF57Δ146, purified selenomethionine (Se-Met) derivatized protein was concentrated to 300 μM in Vivaspin 500 centrifugal devices with a 5 kDa MWCO immediately prior to setting up crystal trials. The hvsORF57Δ146-RNA complex was obtained by mixing of 30 μM native (non Se-Met) protein with 90 μM HPLC purified RNA oligo PAN bases 34–50, sequence CACCUAUGGAUUUUGUG (Sigma) here abbreviated to PAN17, the solution was stored at 4°C for 60 min, then concentrated in a Vivaspin 500 centrifugal device with a 5 kDa MWCO 10-fold prior to crystal trials. Concentrated samples were used to setup 4  ×  96 crystal trials and screened by the sitting drop vapor diffusion method. A 200 nl drop of purified hvsORF57Δ146 or hvsORF57Δ146-RNA were mixed with 200 nl of the screen condition using a TTP Mosquito Crystal nanolitre pipetting robot. Following 24 h incubation at 4°C the plates were manually inspected and single crystals suitable for X-ray diffraction analysis were observed in a range of conditions. Selenomethionine derivatized RNA-free hvsORF57Δ146 SeMet crystals grew from reservoir solutions consisting of 0.12 M Alcohols (0.2 M 1,6-hexanediol; 0.2 M 1-butanol; 0.2 M 1,2-propanediol; 0.2 M 2-propanol; 0.2 M 1,4-butanediol; 0.2 M 1,3-propanediol), 0.1 M (Imidazole, MES) Buffer System pH 6.5, 50% (v/v) GOL_P4K Mix (Morpheus HT96 D3 Molecular Dimensions). hvsORF57Δ146-PAN17 crystals grew from reservoir solutions consisting of 0.06 M divalents (0.3 M magnesium chloride hexahydrate; 0.3 M calcium chloride dehydrate), 0.1 M (Imidazole, MES) buffer system pH 6.5, 50% (v/v) GOL_P4K (Morpheus HT96 A3 Molecular Dimensions). Crystals were flash frozen by plunge freezing in liquid nitrogen prior to data collection at Diamond Light Source Ltd. Identical attempts at producing diffraction quality crystals using ksORF57Δ153 in place of hvsORF57Δ146 in both the presence and absence of PAN17 RNA were carried out without success.

### Data collection, structure determination, model building and refinement

Data were collected from three single cryo frozen crystals. A high redundancy dataset was collected from a single selenomethionine (SeMet) derivatized crystal of free hvsORF57Δ146 at Diamond Light Source (DLS) (i03). This SeMet data was subsequently used to solve the structure by single wavelength anomalous dispersion (SAD). A high-resolution native data set was collected (DLS i03) to a resolution of 1.86 Å and subsequently used to refine and rebuild the ORF57 model. A third crystal was used to collect a native data set of the ORF57 RNA complex hvsORF57Δ146-PAN17 (DLS i04-1).

All data were scaled and merged in Xia2 with phasing performed with the Big EP pipeline (60). The partial model produced from with Big EP was used as the basis for further iterative cycles of model building and refinement in COOT and Phenix.refine respectively. A complete model was rebuilt and refined against the high-resolution 1.86 Å native data. Validation with both MolProbity and PDB_REDO were integrated into the iterative rebuild process (61,62). The hvsORF57Δ146-PAN17 RNA complex structure was determined by molecular replacement in PHASER using a model derived from the native structure hvsORF57Δ146. Automated model building in Phenix Autobuild produced a near complete hvsORF57Δ146-PAN17 model of the protein with clear difference density for the PAN17. Two uridine nucleotides were well ordered in these early maps showing clear stacking interactions with the side chain of Y311. Subsequent manual rebuilding in COOT and refinement cycles in Phenix.refine were implemented to generate the final hvsORF57Δ146-PAN17 model. Complete data collection and refinement statistics are presented in Table 2. Data were deposited into the protein data bank, codes 6HAU and 6HAT for the free and RNA-bound structures, respectively.

### Biophysical characterization of ORF57 globular domains

For size exclusion chromatography coupled with multi angle light scattering (SEC-MALS) analysis, samples (0.5 ml at 1 mg/ml) were loaded onto a Superdex 200 10/300GL column (GE life-sciences, 0.75 ml/min in gel filtration buffer) and passed through a Wyatt DAWN Heleos II EOS 18-angle laser photometer coupled to a Wyatt Optilab rEX refractive index detector. Data were analyzed using Astra 6 software (Wyatt Technology Corp., CA, USA).

### NMR spectroscopy

Prior to NMR studies proteins were dialyzed into NMR buffer: 20 mM sodium phosphate, 50 mM NaCl, 50 mM l-Arg, 50 mM l-Glu, 1 mM EDTA, 2 mM TCEP, pH 6.2 and concentrated using Vivaspin centrifugal devices. 5% (v/v) D_2_O was added to samples for lock and data was acquired at 298 K on a Bruker Avance III 800 MHz spectrometer equipped with a cryoprobe. Topspin 3.2 was used for data acquisition and processing. Mapping of RNA binding sites was carried out by acquisition of HSQC spectra of 50 μM uniformly ^15^N labelled protein without binding partner and then addition of a small volume of unlabeled RNA oligo, either PAN17 (PAN bases 34–50 sequence CACCUAUGGAUUUUGUG) or 14merS (CAGUCGCGAAGAGG), from a concentrated stock in matching buffer resulting in an equimolar mixture of the two species. A second HSQC spectrum of the bound state was then acquired for comparison. The distance of signal movement were measured as described previously for the ICP27-REF^1–155^ interaction (63), signal shifts >1 standard deviation of all peak movement distances (1σ) were judged ‘moderate’ and greater than 2σ ‘large’. Peak heights were also measured and compared between bound and free states, a loss in intensity due to broadening was labelled significant if the signal loss was >75%. All spectral assignment and analysis was carried out using Sparky (64).

### Surface plasmon resonance (SPR)

Purified proteins ksORF57Δ153, hvsORF57Δ146, UL69^200–540^ and HSV-1 ICP27Δ241 were exhaustively dialyzed into buffer B (20 mM HEPES, 150 mM NaCl, 1 mM TCEP, pH 7.4). Experiments were performed using the ProteOn XPR36 SPR instrument (Bio-Rad Laboratories). The ProteOn XPR36 is a multiplex system that can be used to provide simultaneous flow of up to six different analyte concentrations (channels A1–A6) over up to six different ligand channels (L1–L6). Running buffer (RB) was 250 mM NaCl, 20 mM HEPES, 0.05% (w/v) Tween-20, pH 7.4. All experiments were performed at 25°C. Immobilization of biotinylated RNA oligos was performed on a GLC chip (Bio-Rad Laboratories) in the vertical orientation. Four channels (L2, L3) were activated with 150 μl of a 1:1 mixture of 20 mM N-ethyl-N′-(3-dimethylaminopropyl) carbodiimide (EDC) and 6.5 mM sulfo-N-hydroxysuccinimide (sulfo-NHS) in water at a flow rate of 30 μl/min. NeutrAvidin was diluted in 10 mM sodium acetate buffer pH 5 to a final concentration of 50 μg/ml, and 150 μl was injected, followed by an injection of 150 μl of 1 M ethylenediamine–HCl, pH 8.5, at a flow rate of 30 μl/min. The immobilization level of NeutrAvidin was ∼2000 resonance units (RU). Then RNA oligos were bound on channels L2–4 to an immobilization level of ∼100 RU. The RNA oligos used were PAN17 (CACCUAUGGAUUUUGUG) and 14merS (CAGUCGCGAAGAGG) as well as a non-specific control 14mer1 (CCGUCCCCGCCGCU). The L1 channel (no RNA) was used as a reference. Measurements of binding were made using different concentrations of viral protein constructs in channels A2–A6, channel A1 was used as a buffer only control. A short pulse of 2 M NaCl (50 μl/min for 60 s) was used for regeneration between measurements. Non-specific binding of protein to the reference channel precluded the use of analyte concentrations above 1 μM. All binding sensorgrams were collected, processed and analyzed using the integrated ProteOn Manager software (Bio-Rad Laboratories). Short black segments on some sensorgrams represent artefact (spike) removal from the data. Sensorgrams were assessed qualitatively for RNA interactions.

### RNA consensus site analysis

KSHV ORF57 interactions with nine sites on PAN RNA were identified by Sztuba-Solinska *et al.* (53). The RNA sequences detected as ORF57 interaction sites were collated and in addition to the direct binding sites, two flanking bases both 5′ and 3′ of each of the sites were also included in the list (Supplementary Table S1). These were aligned with Clustal omega (65) (Figure 3D). The alignment was then used to generate a WebLogo to illustrate a potential consensus sequence (66).

## RESULTS

### Identification of RNA binding sites within the KSHV ORF57 N-terminal domain

**Figure 1.**
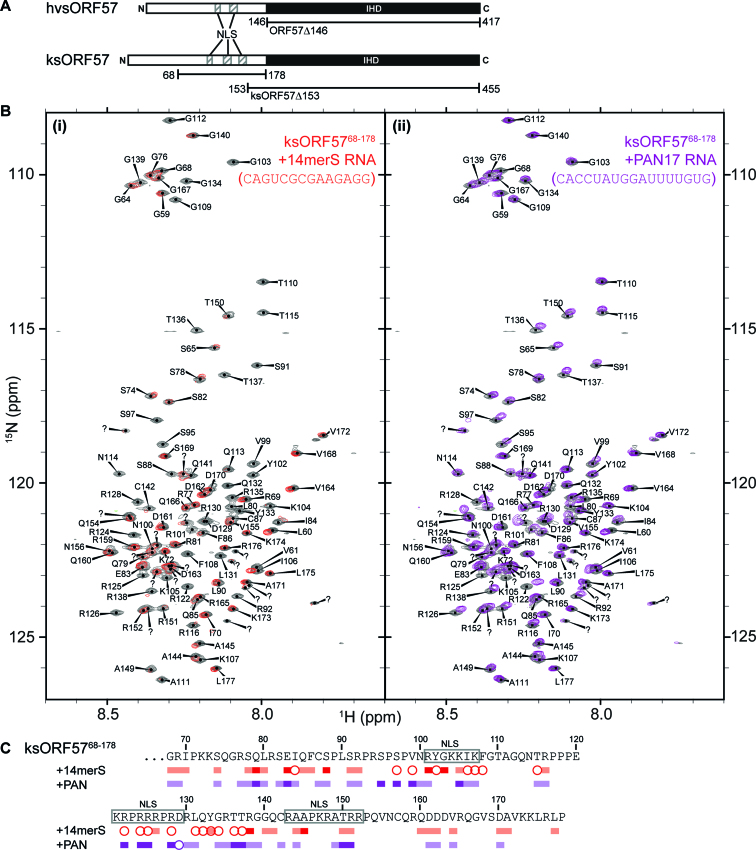
Interaction mapping of RNA with KSHV ORF57 residues 68–178 by NMR. (**A**) Schematic of domain organization within the HVS and KSHV ORF57 proteins, and the boundaries of the constructs used throughout this study. N-terminal intrinsically disordered domain is shown as white bar with positions of nuclear localization sequences (NLS) marked, and C-terminal globular ICP27-homology domain (IHD) is shown as black bar. (**B**) ^15^N HSQC spectra of free protein (colored grey) overlaid with spectra of protein bound with RNA oligos, (i) 14merS oligo (colored red) or (ii) PAN17 oligo (colored purple). (**C**) Signal shifts and intensity perturbations induced by RNA oligos mapped onto the sequence of ksORF57 from analysis of spectra in panel A. Residues with signals broadened are marked by circles, and moderate and large signal shifts indicated by light and dark blocks, respectively. The positions of three NLS motifs are marked.

We have previously used NMR spectroscopy to determine an RNA binding site within HVS ORF57 N-terminal residues 8–120 (hvsORF57^8–120^), an intrinsically disordered region (57). The data indicated preferential binding to an oligo containing a GAAGRG sequence motif by residues 64–120; sequence comparison suggested an analogous region (with 21% identity) within KSHV ORF57, namely residues 79–145. To investigate the RNA binding properties of the N-terminal domain of ksORF57 we utilized a construct comprising residues 68–178 (ksORF57^68–178^), which contained the region analogous to the hvsORF57^8–120^ RNA binding site plus flanking sequences containing three NLSs also implicated in RNA interactions in experiments by Majerciak *et al.* (26) (Figure 1A). In order to investigate RNA specificity within this region of ksORF57 we used two RNA oligos: first, a 14mer oligo (14merS) previously used in HVS ORF57 RNA binding experiments containing the GAAGRG motif (56,57), and second, a 17mer oligo (PAN17) comprising PAN RNA bases 34–50, which form a loop region in the MRE implicated in ksORF57 interactions (27,50,53). HSQC spectra of a ^15^N-labelled sample of ksORF57^68–178^ were acquired in the presence and absence of a stoichiometric amount of an unlabeled RNA oligo, either PAN17 or 14merS. The addition of 14merS induced substantial signal broadening throughout ksORF57^68–178^. In contrast, the PAN17 RNA only induced moderate broadening and shifts in signal positions (Figure 1B). We have previously observed similar signal perturbation patterns in hvsORF57^8–120^, which interacts specifically with the GAAGRG motif within the 14merS oligo resulting in substantial broadening, while a non-specific lower affinity oligo only caused moderate perturbations (57). Therefore the data was suggestive of specificity within ksORF57^68–178^ for the 14merS RNA oligo and likely the GAAGRG motif, and weaker non-specific interaction with the PAN17 RNA. The backbone signal assignments of ksORF57^68–178^ (BMRB entry 27484 (39)) allowed mapping of the RNA binding site, and the major perturbations were observed in the region containing residues 79–145 (Figure 1C). Also in common with HVS ORF57, part of the RNA binding region overlaps an ALYREF binding site on KSHV ORF57 mapped to residues 126–134 (39). The data therefore suggested that the N-terminal domains of HVS ORF57 and KSHV ORF57 have a conserved binding preference for RNA fragments containing the purine-rich GAAGRG motif, and significantly, the ksORF57^68–178^ construct only binds weakly with the PAN17 RNA oligo.

### RNA interaction screening by surface plasmon resonance

As the interaction of the PAN17 oligo with the N-terminal domain of ksORF57 appeared non-specific, we hypothesized that another region of ORF57 might recognize this RNA. We therefore investigated if the C-terminal globular ICP27-homology domains (IHD) of the closely-related ORF57 proteins from KSHV (ksORF57) and HVS (hvsORF57) interacted with reporter RNAs, and in parallel we also carried out the same experiments with the distantly-related homologous IHDs from HSV-1 ICP27 and HCMV UL69. The regions containing the IHD of HSV-1 ICP27, HCMV UL69 and ksORF57 have already been determined (42,43,48), but the position of the folded domain within hvsORF57 was not yet defined. Therefore sequence alignments along with secondary structure and disorder predictions were carried out with hvsORF57, which predicted that residues 146–417 comprised the IHD. A protein construct comprising this region (hvsORF57Δ146) was cloned, then expressed in *E. coli* and purified to homogeneity. Similarly the proteins ICP27Δ241, UL69^200–540^ and ksORF57Δ153 were also purified (39,43,48) (Supplementary Figure S2). Surface plasmon resonance (SPR) experiments were performed using three 5′-biotinylated RNA oligos (Sigma) with the sequences of PAN17, 14merS and 14mer1 (the latter a negative control), which were immobilized onto separate channels on a NeutrAvidin coated sensor chip. Proteins were then flowed over the sensor and the response relative to a NeutrAvidin control channel without RNA was monitored. RNA interactions were rated as absent, moderate or strong and summarized in Table 1 (raw data in Supplementary Figure S2). UL69^200–540^ was promiscuous for interactions with RNAs whereas ICP27Δ241 failed to significantly interact with any of these RNAs. Significantly, the hvsORF57Δ146 and ksORF57Δ153 proteins both preferentially interacted with the PAN17 RNA, suggesting a probable specific RNA binding site within the ORF57 C-terminal IHDs.

**Table 1.** Surface plasmon resonance binding screen

	RNA oligo		
Protein	PAN17	14merS	14mer1
ICP27Δ241	–	–	–
ksORF57Δ153	+	±	–
hvsORF57Δ146	+	±	–
UL69 ^200–540^	+	+	+

Qualitative analysis of sensorgrams indicated and absence (−), weak (±) or appreciable (+) interaction of IHD protein constructs with different RNA oligos.

### The C-terminal IHDs from hvsORF57 and ksORF57 form homo-dimers in solution

**Figure 2.**
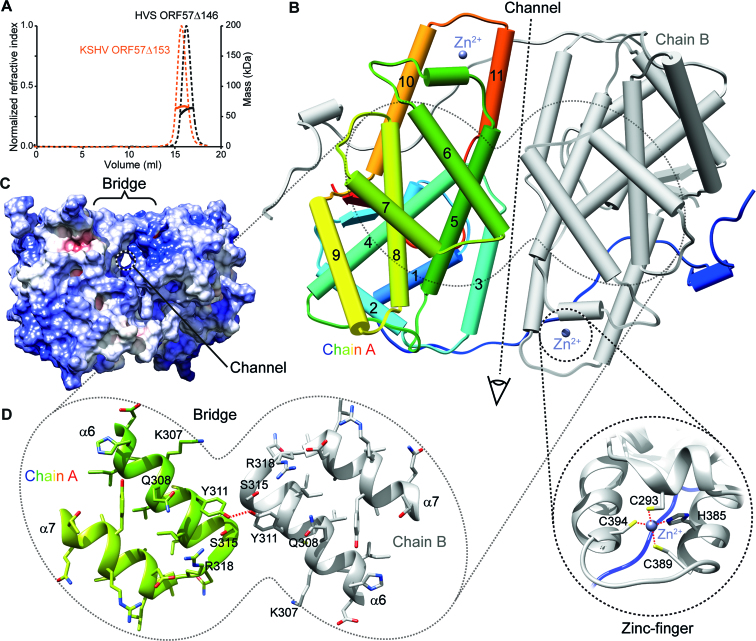
The structure of the C-terminal IHD from HVS ORF57. (**A**) Analysis of oligomeric state of IHD constructs hvsORF57Δ146 and ksORF57Δ153 by SEC-MALS indicated homo-dimerization. Size-exclusion chromatogram of purified proteins, dashed lines indicate refractive index with scale on left axis, solid lines represent molar mass quantified with scale on right axis. (**B**) X-ray crystal structure of the hvsORF57Δ146 homo-dimer. Chain A is colored blue through red from N- to C-termini with α-helices labelled and shown as cylinders, while chain B is colored grey. CHCC Zinc-finger locations are indicated by the spheres marked Zn^2+^ and the lower insert shows detail this feature. (**C**) Protein surface view colored by electrostatic potential in an orthogonal orientation to panel B, as indicated by eye symbol. A narrow channel through the protein passing under helix α6 is apparent. (**D**) Detail of helices α6 and α7 that form the homo-dimer bridge feature. Colored as panel A with sidechain atoms shown as sticks; selected residues are labeled. Red dash marks hydrogen bond between the Y311 sidechains.

A dimeric state of KSHV ORF57 was previously indicated by cross-linking experiments (42), and later both monomer and dimer states were observed in crystal structures (49). Therefore we characterized the solution oligomeric state of purified hvsORF57Δ146 and ksORF57Δ153 proteins by SEC-MALS. The predicted monomeric molecular weights by primary sequence of hvsORF57Δ146 and ksORF57Δ153 are 30.7 and 33.9 kDa, and we measured 61.5 and 66.1 kDa respectively. The data therefore indicated that both ORF57 constructs eluted with a molecular weight indicative of a stable homo-dimer (Figure 2A).

### Structure determination of the C-terminal IHD of hvsORF57

In order to investigate the 3D structure of an ORF57 C-terminal IHD experimentally, first we purified the hvsORF57Δ146 and ksORF57Δ153 protein constructs in the absence of RNA and screened with a variety of crystallization conditions using six commercial screens (SG1, JCSG+, PACT Premier, BCS, LMB and Morpheus; Molecular Dimensions). However only the hvsORF57Δ146 construct produced diffracting crystals. Initial phases were obtained from single wavelength anomalous dispersion (SAD) experiments carried out on selenomethionine derivatized crystals in Phenix.AutoSol (67). Model building and refinement were carried out against the same data, extending to a resolution of 1.86 Å (Table 2). Complete details of the structure determination and model building can be found in the Materials and Methods section. The structure (Figure 2B) revealed two molecules of hvsORF57 in the asymmetric unit with clearly interpretable electron density for all residues 145–417 of chain A and 147–417 of chain B. Data were deposited into the protein data bank (code: 6HAU).

**Table 2.** Crystallography refinement statistics for hvsORF57Δ146 free and RNA complex

	Free ORF57*	ORF57-PAN17*
Wavelength	0.9763	0.9282
Resolution range	48.47–1.86 (1.92–1.86)	78.71–1.86 (1.93–1.86)
Space group	*P* 2_1_ 2_1_ 2_1_	*P* 2_1_ 2_1_ 2_1_
Unit cell	58.1 118.7 130.7 90 90 90	58.7 105.3 118.6 90 90 90
Total reflections	1 027 470 (100 958)	469 581 (47 523)
Unique reflections	77 002 (7497)	62 577 (6135)
Multiplicity	13.3 (13.5)	7.5 (7.7)
Completeness (%)	99.83 (98.48)	99.89 (99.79)
Mean *I*/sigma(*I*)	18.04 (2.55)	18.23 (2.67)
Wilson *B*-factor	32.87	28.14
*R*-merge	0.083 (0.97)	0.073 (0.72)
*R*-meas	0.087 (1.01)	0.079 (0.78)
*R*-pim	0.024 (0.27)	0.028 (0.28)
CC_1/2_	0.99 (0.81)	0.99 (0.80)
CC*	1 (0.95)	1 (0.942)
Reflections used in refinement	76996 (7496)	62543 (6135)
Reflections used for R-free	2000 (195)	3127 (333)
*R*-work	0.150 (0.257)	0.158 (0.252)
*R*-free	0.169 (0.257)	0.197 (0.266)
CC(work)	0.967 (0.884)	0.962 (0.902)
CC(free)	0.958 (0.911)	0.944 (0.859)
Number of non-hydrogen atoms	4727	5002
Macromolecules	4373	4565
Ligands	10	6
Solvent	344	431
Protein residues	544	546
RMS(bonds)	0.014	0.009
RMS(angles)	1.44	1.26
Ramachandran favored (%)	98.70	98.15
Ramachandran allowed (%)	1.30	1.85
Ramachandran outliers (%)	0.00	0.00
Rotamer outliers (%)	0.79	0.78
Clashscore	1.68	2.49
Average *B*-factor	45.50	37.74
Macromolecules	45.18	37.36
Ligands	59.39	49.56
Water	49.18	41.52
Number of TLS groups	3	3

*Highest resolution shell is shown in parenthesis.

### Structural features of ORF57

The C-terminal IHD of hvsORF57 forms a compact homo dimer, approximately 65  ×  60  ×  40 Å. Each polypeptide chain is composed of 11 α-helices and a small β-sheet formed by two parallel strands, folded into an individual globular domain (Figure 2B). One CHCC zinc finger is formed by each chain from residues C293, H385, C389 and C394, which coordinate a zinc ion in a tetrahedral geometry. Residues 181–417 form the globular folded core domain preceded by residues 146–180, which comprise an extended N-terminal arm largely lacking secondary structure that domain swaps, wrapping around the dimeric partner's globular domain. Numerous intermolecular contacts are made between the two protein chains. PDBePISA analysis (68) indicated that 34% of residues observed in the structure have atoms within the dimer interface. The major homo-dimer interface is linear and formed by contacts between α-helices 3, 5 and 11 which lie approximately parallel to each other making symmetric contacts and forming a groove. The groove is bridged by α-helix 6, where residues Y311 and S315 from separate polypeptide chains contact each other (Figure 2B–D), with the sidechain hydroxyl oxygens of the Y311 pair distanced by 2.6 Å. A hydrated narrow channel is present beneath the bridge with the dimeric pair of H285 sidechains at the center, at this point the width of the channel is narrowed to ∼5.5 Å. Analysis of the surface electrostatic distribution indicated that the groove has a net positive charge (Figure 2C).

### Structural determination of the RNA complex with the C-terminal IHD of HVS ORF57

**Figure 3.**
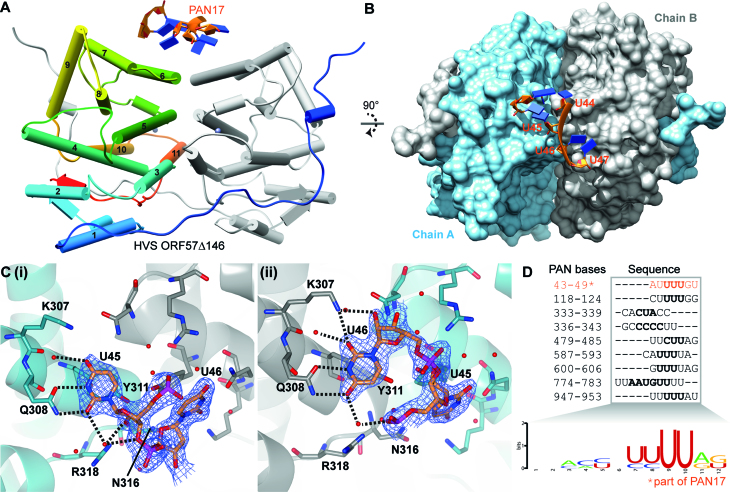
PAN RNA MRE U-bend motif interaction with hvsORF57Δ146. (**A**) Structure of PAN17 interaction with hvsORF57Δ146: polyuridine RNA interacts with the bridge formed by helix α6 at the homo-dimer interface. RNA is colored orange and blue on backbone and bases respectively, protein chain A is colored rainbow blue through red from N- to C-termini, chain B is shaded grey. (**B**) Orthogonal view of panel A showing protein surface colored cyan and grey for chains A and B respectively. (**C**) Detail of protein-RNA recognition of uridine bases at the homo-dimer interface. RNA bases U45 and U46 plus protein sidechains that contact RNA are shown as sticks and labelled, water molecules shown as red spheres. Hydrogen bonds indicated by black dashes. The two uridine recognition sites formed by each protein chains: (i) Chain A interaction with U45, (ii) Chain B interaction with U46. Electron density assigned to RNA is shown as blue mesh scaled to 1.5σ. (**D**) The nine sites on PAN RNA which KSHV ORF57 interacts identified by Sztuba-Solinska *et al.* (53) contain polyuridine sequences which allow generation of a consensus binding sequence logo. The direct interaction with 43–49 site marked with asterisk is observed in the structure presented on the left.

**Figure 4.**
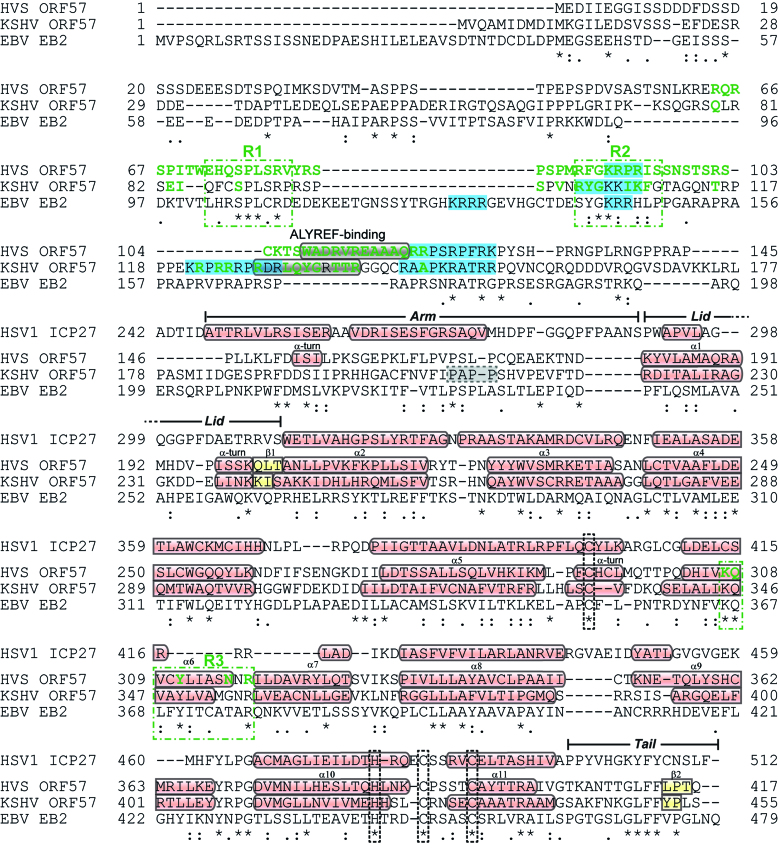
Sequence alignment and summary of binding motifs of ORF57 homologs, in comparison with HSV1 ICP27. The alignment was generated by Clustal omega (65) for HVS ORF57, KSHV ORF57 and EBV EB2 sequences, whereas the C-terminal IHD sequence of HSV 1 ICP27 was aligned with HVS ORF57 to match position of structural elements. Secondary structure elements for HVS, KSHV and ICP27 are marked, with α-helices and β-sheets colored red and yellow, respectively. NLS are highlighted in light blue. The PxxP motif of ksORF57 implicated in ALYREF interactions is marked by a grey dashed box (30). Residues colored green are located in RNA binding sites in HVS and KSHV ORF57 and were identified by NMR or X-ray crystallography data, with conserved sites in γ-herpesviruses marked by dashed green boxes and labelled R1, R2 and R3. Conserved zinc coordinating residues are marked by black dashed boxes.

To investigate PAN RNA binding to ORF57 from the structural perspective, purified hvsORF57Δ146 mixed with a 3-fold molar excess of a 17-mer RNA oligo matching bases 34–50 of PAN (PAN17) was screened against crystallization conditions, and produced diffracting crystals. The hvsORF57Δ146-PAN17 diffraction data was solved by molecular replacement with Apo hvsORF57Δ146 coordinates. Data were deposited into the protein data bank (code 6HAT). Continuous electron density was assignable to residues 145–417 in both ORF57 polypeptide chains that formed a homodimer (residue 145 is a non-WT glycine introduced in cloning). The conformation of hvsORF57Δ146 was unchanged from that observed in the Apo form, with a Cα RMSD of 0.6 Å (residues 147–417). The RNA oligo was observed in association with the bridge formed by the two α6-helices at the dimer interface, and the electron density was assigned to the bases 44–47 of PAN, a UUUU sequence, plus an isolated nucleotide pair assigned to the 5′ CA sequence of the PAN17 oligo (Figure 3A,B). Within the uridine-quartet sequence, the second nucleotide binds to protein chain A while the third binds chain B, with the linking ribose-phosphate bridging the protein homo-dimer. Both the second and third uracil bases contact the same amino acid residue numbers but in different chains. Specifically the PAN RNA bases U45 and U46 form a π-stacking interaction above the sidechain of Y311, a pair of hydrogen bonds with the sidechain of Q308, plus one hydrogen bond with a water molecule, which is also coordinated by the sidechain amine of K307 (Figure 3C). R318 forms stabilizing ionic bonds with phosphates of the RNA backbone. The planes of the aromatic Y311 sidechains are angled approximately perpendicular to each other and therefore the U45–U46 bases that they bind are complementarily angled with a tilt of –95° and twist of 112°, the RNA therefore is bent in order to facilitate this protein–RNA contact. The contacts of hvsORF57 with the other bases do not appear to provide obvious base selectivity. The first and fourth nucleotides of the UUUU sequence are positioned above and forming stacking interactions with the second and third bases (Figure 3B), the 5′ cytosine base also makes a base stacking interaction with the first uracil of the quartet and a single non-Watson–Crick hydrogen bond with the first U. The uridine-quartet fills four positions that comprise an ORF57 C-terminal IHD RNA interaction site, with other nucleotides forming additional apparently non-specific protein contacts. The four residues K307, Q308, Y311 and R318 of hvsORF57 that contact the PAN RNA oligo are conserved in ksORF57 and the Epstein-Barr virus (EBV) homolog EB2 (R3 site, Figure 4). Uniquely the four RNA interacting residues are the only conserved surface exposed amino acids within the hvsORF57 structure, except for an isolated R399 which is adjacent to the zinc coordination site.

The structure of the C-terminal IHD RNA binding site is suggestive of binding preference for pyrimidine and likely uridine bases, and the bent conformation of the RNA as it contacts ORF57 is indicative that the nucleotides should be flexible and not constrained by base-pairs. We therefore use the term ‘U-bend’ to describe this RNA motif. Considering steric factors, a cytosine base could also be accommodated in place of the uracil, however when the hydrogen bond patterns are also considered this would not be compatible in the site on chain B and only possible in chain A if the sidechain amide of Q308 adopts a different rotamer with the sidechain amide flipped 180 degrees. The steric bulk of larger purine bases could not be accommodated in the conformation observed. In the structure, any specificity determinants for the peripheral bases were not discernible, therefore the structure is suggestive of a single stranded RNA binding motif compatible with NYUN, where N is any base and Y is a pyrimidine. As an indirect probe for specificity, we were able to crystallize hvsORF57Δ146 in the presence of both shorter RNA oligos corresponding to sub-fragments of PAN17, namely CACCUAU and GAUUUUGU (PAN 34–41 and PAN 42–49 respectively), however electron density for RNA was only discernible in the latter, which matched the same U-bend interaction observed for PAN17 (data not shown).

### Analysis of a KSHV ORF57 interaction motif within PAN RNA

In light of the apparent uracil specificity observed in the hvsORF57-PAN17 structure, and conservation of the binding site in KSHV ORF57 we looked for evidence in the literature of additional U-bend-like sites. Notably, a recent study by Sztuba-Solinska *et al.* (53) identified nine KSHV ORF57 binding sites on KSHV PAN RNA and the majority contained poly-uracil sequences. Sequence alignment of the sites allowed production of a preliminary consensus motif logo (66) (Figure 3D). These previous data therefore correlated with our structural and sequence conservation results suggestive of a conserved uridine-specific binding site on the C-terminal IHDs of ORF57 homologs in HVS, KSHV and EBV.

### Comparison of ORF57 and ICP27 structures

**Figure 5.**
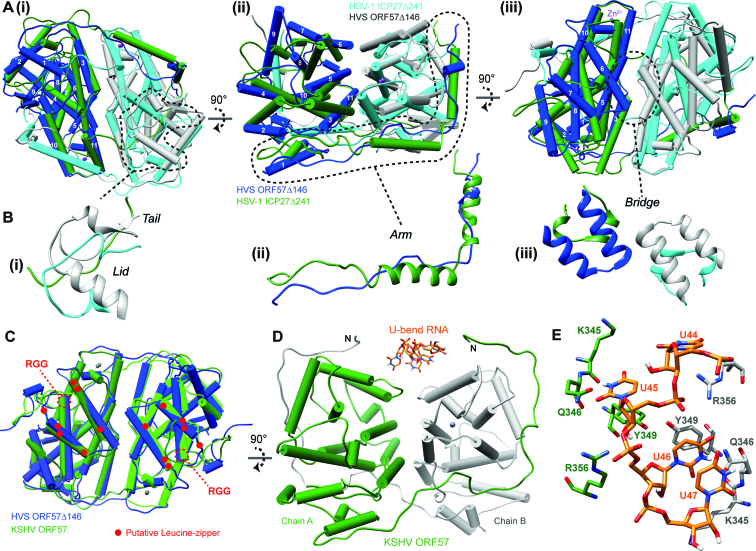
Comparison of the ICP27-homology domain structures. (**A**) Orthogonal views of a superposition of the X-ray crystal structures of hvsORF57Δ146 (colored blue and grey for chains A and B respectively) and HSV-1 ICP27Δ241 (colored green and cyan for chains A and B respectively). Dashed lines mark structural motifs shown in detail in panel (**B**). (**C**) Superposition of hvsORF57Δ146 (chains A and B colored blue and light blue respectively) with KSHV ORF57 homo-dimer coordinates, PDB: 5ZB3 (chains A and B colored green and light green respectively). The locations of ksORF57 motifs previously targeted by mutations are labelled: the isolated RGG sequences are marked with dashes and regions previously referred to as Leucine-zippers are marked by red dots. (**D**) The PAN17 RNA U-bend coordinates from the structure determined here (shown in stick representation) are docked onto the conserved binding surface of ksORF57 (colored green and grey for chains A and B respectively) (E) Detail of the docked PAN17 RNA poly-uridine sequence interaction with the ksORF57 coordinates, colored as in panel D. Generated by UCSF Chimera (73).

Comparison of the conformation of hvsORF57Δ146 with the structure of the homologous C-terminal IHD region from HSV-1 ICP27 (residues 241–512), regions that share 19% sequence identity, indicates they clearly adopt the same core protein fold (Figure 5A) and the globular regions comprise a core α-helical bundle with Cα RMSD of 4.8 Å (when aa276–499 and aa181–404 of ICP27 and hvsORF57, respectively, were overlaid). The Cα RMSD was 12.4 Å for the complete protein chains, which was indicative of a number of differences. The C-terminal ‘tail’ of ORF57 containing the ‘GLFF motif’ makes intramolecular contacts in the form of a short parallel β-sheet with residues 200–204, whereas in ICP27 the C-terminal tail domain swaps and is covered by ‘lid’ formed by a loop from its dimeric partner (Figure 5B,i). The intramolecular contact observed in the C-terminal tail of hvsORF57 indicates that domain-swapping of this region observed previously in ICP27 is not a ubiquitous feature essential for dimerization of all proteins with an IHD fold. Outside of the globular core, the N-terminal arm of hvsORF57 is composed of an α-helical turn and an extended loop contacting the dimeric partner. This lack of secondary structure is in contrast with two α-helices that form the arm of ICP27 (Figure 5B,ii). The homo-dimerization interface region is groove shaped in ICP27, whereas in hvsORF57 the groove is bridged by helices α6, forming the U-bend binding site (Figure 5B,iii). As the bridge feature is not present in the structure of ICP27, the structure therefore correlates with the lack of interaction of ICP27 with the RNA oligos used in our SPR experiment. Sequence comparison of hvsORF57 with HCMV UL69^200–540^ indicated no conservation of the U-bend binding site (Supplementary Figure S3), and therefore the location of the promiscuous RNA oligo interaction sites within the UL69 tetramer remain unknown.

### KSHV ORF57 structure analysis

A low-resolution (3.5 Å) structure of ksORF57 (Protein Data Bank code 5ZB3) was recently published which revealed a dimeric fold with a domain swap mediated by an N-terminal arm (49). The ksORF57 structure closely resembled that of hvsORF57 with near-identical secondary structure composition (Figure 4) and superposition of the dimers indicating a Cα RMSD of 2.6 Å, calculated for both chains of the homo-dimer residues using residues 147–417 hvsORF57 (Figure 5C). The combined hsvORF57 and ksORF57 data allowed the location of sequence features previously probed in mutagenesis experiments to be mapped on the structure of the folded domain: the RGG sequence (41) is located at the start of helix α8, while the region previously referred to as Leucine-zipper does not form a zipper, instead forming helices α6 and α7, which form the bridge feature (Figure 5C). The U-bend interaction site (R3, see Figure 4) formed by a pair of helices α6 and the adjacent loops, is conserved and the RNA coordinates from hvsORF57Δ146-PAN17 complex could be docked onto the ksORF57 structure in the same location (Figure 5D). This indicated PAN RNA bases U45 and U46 contact four conserved ksORF57 residues K345, Q346, Y349 and R356.

## DISCUSSION

The ICP27 homolog expressed by Kaposi's sarcoma herpesvirus (KSHV) named ORF57 (ksORF57) promotes the stabilization of a functionally important long non-coding polyadenylated (PAN) RNA as well as other nascent transcripts (23,24,28,29,34). While RNA regions bound by ksORF57 have been established, such as the PAN RNA Mta-responsive element (MRE) (27,50), and RNA binding sites on the ksORF57 protein implied via mutagenesis experiments (26,41), details of their specificities and precise locations have been lacking. Therefore in order to gain a new level of detail of these crucial interactions we have used structural biology techniques to resolve RNA binding sites within ksORF57 and which provided new insights into their RNA specificities.

The structure of the ksORF57 protein is composed of an N-terminal intrinsically disordered region and a globular C-terminal ICP27-homology domain. We used the construct ksORF57^68–178^ to identify RNA interaction sites within the N-terminal domain using NMR signal perturbation mapping, which indicated residues 79–145 bind RNA with an apparent preference for an oligo containing the sequence motif GAAGRG over a PAN MRE derived oligo (Figure 1). Specificity for a GAAGRG motif within ksORF57 has not been previously identified, however this motif is known to bind the N-terminal domain within the ORF57 homolog from herpes virus saimiri (hvsORF57) (56,57). Sequence comparison of the hvsORF57 and ksORF57 N-terminal domains indicated regions of conservation which, along with a poorly conserved ALYREF binding site (39), encompass the RNA interacting regions identified by NMR in both proteins. The data therefore suggested ksORF57 residues 86–92 and 99–108 (labelled R1 and R2 respectively in Figure 4), plus part of an ALYREF binding site (residues 126–134) form a RNA binding region, with a preference for the purine-rich motif GAAGRG over a PAN RNA MRE fragment.

The C-terminal globular IHD of ksORF57 has previously been implicated in RNA interactions (28,41,42); we therefore used surface plasmon resonance to screen for RNA binding within the ksORF57Δ153 construct in parallel with IHD-containing regions of the proteins hvsORF57, HSV-1 ICP27 and HCMV UL69. The γ-herpesvirus close homologs hvsORF57 and ksORF57 both displayed similar responses with the three oligos used, indicative of a preference for the PAN MRE (PAN17). HCMV UL69 bound with all RNA oligos, while in contrast HSV-1 ICP27 did not interact significantly (Table 1, Supplementary Figure S2). We therefore attempted to determine the structure of the PAN MRE-ORF57 complex by X-ray crystallography; while we were unable to obtain ksORF57 data we did solve the hvsORF57 C-terminal IHD structure in both the presence and absence of a PAN17, both at 1.86 Å resolution. The structure revealed an RNA interaction site located on a bridge feature at the homodimer interface, formed by a pair of α6 helices, one from each monomer (Figures 2 and 3). The RNA-interacting residues are conserved between HVS and KSHV ORF57 proteins plus the homolog EB2 from EBV (marked R3 in Figure 4). The data indicated an apparent binding specificity for uridine nucleotides in a bent, non-base paired conformation; the RNA motif was therefore named the U-bend (Figure 3).

Comparison of the ICP27 C-terminal IHD structure and sequence with hvsORF57 clearly indicated the U-bend binding site (i.e. the bridge feature) is not present in ICP27 (Figures 4 and 5A, B) which correlated with paucity of RNA binding in the SPR screen within this domain. In contrast, superposition of the recently determined dimeric ksORF57 apo-structure (49) with our hvsORF57 data indicated conservation of the overall protein fold and, significantly, a near-identical architecture within the U-bend binding site. Sequence conservation and structure homology therefore implicate ksORF57 residues K345, Q346, Y349 and R356 as responsible for forming uracil-specific contacts in PAN RNA interactions (Figure 5E). Interestingly, the N-terminal arm within the ksORF57 data is extended from L175 (49) and located adjacent to the U-bend binding site (Figure 5D). If this conformation is maintained in solution, it would place the N-terminal RNA binding sites mapped here in proximity to an extended RNA chain, likely providing multiple protein-RNA contact sites. Previous studies have been carried out with mutations within the ksORF57 C-terminal IHD and revisiting these data provides support to the identified U-bend binding site. Specifically, point mutants of a putative leucine-zipper now known as helices α6 and 7 (Figure 5C) likely disrupted the U-bend binding site architecture and impaired dimerization, which led to the failure to accumulate a target viral mRNA encoding ORF59 and PAN RNA (28,41,42). While RNA interactions with the C-terminal IHD were implied from these previous studies, a positive identification of RNA binding to globular domains of ksORF57 or other homologs was not reported previously. The data presented here therefore provides the first direct evidence of direct RNA binding by globular ICP27-homology domains, and reveals the structural determinants of their sequence specificity.

The significance of the ORF57 U-bend binding site likely reaches beyond just the PAN RNA MRE studied here as several other ksORF57 interaction sites on PAN RNA were identified by Sztuba-Solinska *et al.* (53), which were mainly U-rich (Figure 3D). We therefore hypothesize that the U-bend binding site is utilized in these multiple interactions with ksORF57 and thus contributes to PAN RNA stability. As the cellular nuclear exosome targeting (NEXT) complex protein RBM7 targets U-rich sequences (69), we speculate that ksORF57 may complete for the same RNA binding sites as RBM7. Similarly, ALYREF can compete with the exosome cofactor, hMTR4, to determine transcript fate (70). Therefore the synergistic action of a ksORF57-ALYREF-RNA ternary complex could be a potent competitor with the exosome preventing RNA degradation, contributing to stabilizing effect of ALYREF previously observed (40). The ORF57 C-terminal IHD interaction with RNA likely contributes to other functions in addition to RNA stabilization, due to the abundance of U-rich sequences, for example polypyrimidine sites within introns, which ORF57 may interact with in tandem or competition with spliceosome components to modulate splicing (71,72). The differing purine and pyrimidine binding specificities with the neighboring domains of ORF57 could also provide the protein an ability to modulate RNA structure, by binding to complementary strands and stabilizing single stranded polynucleotides in favor of base-paired conformations. Overall, the synergistic action of the folded and unstructured domains of ORF57 proteins to recognize U-bend and GAAGRG-containing motifs, respectively, is anticipated to convey significant overall specificity for viral RNAs. Thus the ICP27-homology domain and associated disordered regions mediate target specific post-transcriptional control via a conserved yet structurally diverse protein fold, which allow it to perform functions critical to herpes viral lytic replication.

## Supplementary Material

Supplementary Data
