# Supplementary material for: Structural identification of conserved RNA binding sites in herpesvirus ORF57 homologs: implications for PAN RNA recognition
Source: Nucleic Acids Res. 2018 Nov 20;47(4):1987–2001. doi: 10.1093/nar/gky1181 (PMC6393246; doi:10.1093/nar/gky1181)
Supplement: Supplementary Data [file gky1181_supplemental_files.pdf]

Supporting information

Structural identification of conserved RNA binding sites in herpesvirus ORF57 homologs: Implications for PAN RNA recognition

Richard B. Tunnicliffe[1], Colin Levy[1], Hilda D. Ruiz Nivia[2], Rozanne M. Sandri-Goldin[3], Alexander P. Golovanov[1,*]

[1] Manchester Institute of Biotechnology, School of Chemistry, Faculty of Science and Engineering, The University of Manchester, Manchester M1 7DN, UK.

[2] Biomolecular Analysis Core Facility, Faculty of Biology, Medicine and Health, University of Manchester, M13 9PT, UK.

[3] Department of Microbiology and Molecular Genetics, School of Medicine, University of California, Irvine, CA 92697-4025, USA.

* To whom correspondence should be addressed. Tel: +44 161 306 5813; Email: a.golovanov@manchester.ac.uk.

Table S1. PAN RNA interaction sites for KSHV ORF57 identified by Sztuba-Solinska et al. 2017.(1) Binding sites sequences are underlined with 2 flanking bases either site, those involved in base-pairing are italicized.

PAN sequence	Sequence	Is site base paired?
43-49 (NB in MRE and PAN17)	AUUUUGU	No
118-124	CUUUUG*G*	No
333-339	*CACUACC*	Partially
336-343	GCCCCCUU	No
479-485	UUCUUAG	No
587-593	*CAUU*UUA	Partially
600-606	*GUUUUAG*	Partially
774-783	UUAAU*GUUUU*	Partially
947-953	UUUUUAU	No

(A)

```
KSHV      -SPRFDDSIIPRHHGACFNVFIPAPPSHVPEVFTDRDITALIRAGGKDDELINKKISAKK 245
HVS       -LKLFDISILPKSGEPKLFLPVPSLPCQEAEKTNDKYVLAMAQRAMHDVPISSKQLTANL 206
              ** **:*:      : : :*: *.:  *   .*: : *: :  . :*   : .*:::*:

KSHV      IDHLHRQMLSFVTSRHNQAYWVSCRRETAAAGGLQTLGAFVEEQMTWAQTVVRHGGWFDE 305
HVS       LPVKFKPLLSIVRYTPNYYYWVSMRKETIASANLCTVAAFLDESLCWGQQYLKNDFIFSE 266
          :    .: :**:*     *   **** *:** *:..* *:.**::*.: *.*   :::.   *.*

KSHV      KDIDIILDTAIFVCNAFVTRFRLLHLSC--VFDKQSELALIKQVAYLVAMGNRLVEACNL 363
HVS       NGKDIILDTSSALLSQLVHKIKMLPF-CHCLMQTTPQDHIVKQVCYLIASNNRILDAVRY 325
          :. ******:  :  . :* ::::* . *   :::.  :   ::***.**:* .**:::* .

KSHV      LGEVKLNFRGGLLLAFVLTIPGMQSRRSISARGQELFRTLLEYYRPGDVMGLLNVIVMEH 423
HVS       LQTSVIKSPIVLLLAYAVCLPAAIICTKNETQLYSHCMRILKEYRPGDVMNILHESLTQH 385
          *     ::     ****:.: :*.      . .::  .     :*: *******.:*:  : :*

KSHV      HSLCRNSECAAATRAAMGSAKFNKGLFFYPLS 455
HVS       LNKCPSSTCAYTTRAIVGTKANTTGLFFLPTQ 417
           . * .* ** :*** :*:    ..**** * .
```

(B)

```
KSHV      SPRFDDSIIPRHHGACFNVFIPAPPSHVPEVFTDRDITALIRAGGKDDELINKKISAKKI 246
HSV1      --------------------------------------------------------ADTI 245
                                                                  *..*

KSHV      DHLHRQMLSFVTSRH-----NQAYWVSCRRETAAAGGLQTLGAFVEEQMTWAQTVVRHGG 301
HSV1      DATTRLVLRSISERAAVDRISESFGRSAQVMHDPFG----GQPFPAANSPWAPVLAGQGG 301
          *   * :*  ::.*      .:::  *.:      *       *   :  **  .:. :**

KSHV      WFDEKDIDII-------------------------------------------------- 311
HSV1      PFDAETRRVSWETLVAHGPSLYRTFAGNPRAASTAKAMRDCVLRQENFIEALASADETLA 361
           ** :   :

KSHV      ------------------LDTAIFVCNAFVTRFRLLHLSCVFDKQSELALIKQVAYLVAM 353
HSV1      WCKMCIHHNLPLRPQDPIIGTTAAVLDNLATRLRPF-LQCYLKARGLC------------ 408
                            :.*:   *  : :.**:* : *.* :. :.

KSHV      GNRLVEACNLLGEVKLNFRGGLLLAFVLTIPGMQS---RRSI-----SARGQELFRTLLE 405
HSV1      --GLDELCSRRLADIK----DIASFVFVILARLANRVERGVAEIDYATLG-VGVGEKMH 461
             * * *.    ..::     : :**:.* .  :    .*.:     :: *    .   :.

KSHV      YYRPGDVMGLLNVIVMEHHSLCRNSECAAATRAAMGSAKFNKGLFFYPLS-- 455
HSV1      FYLPGACMAGLIEILDTHRQECSSRVCELTASHI-VAPPYVHGKYFYCNSLF 512
          :* **  *. *  *:  *:. *  .  *  ::     :  : :* :**  *
```

Figure S1. Alignment of KSHV ORF57 primary sequence with other IHDs of known structure. (A) With HVS ORF57, indicative of 30% identity with minimal gaps. (B) With HSV-1 ICP27, indicative of 16% sequence identity and large gaps. The CHCC zinc coordinating residues are highlighted yellow.

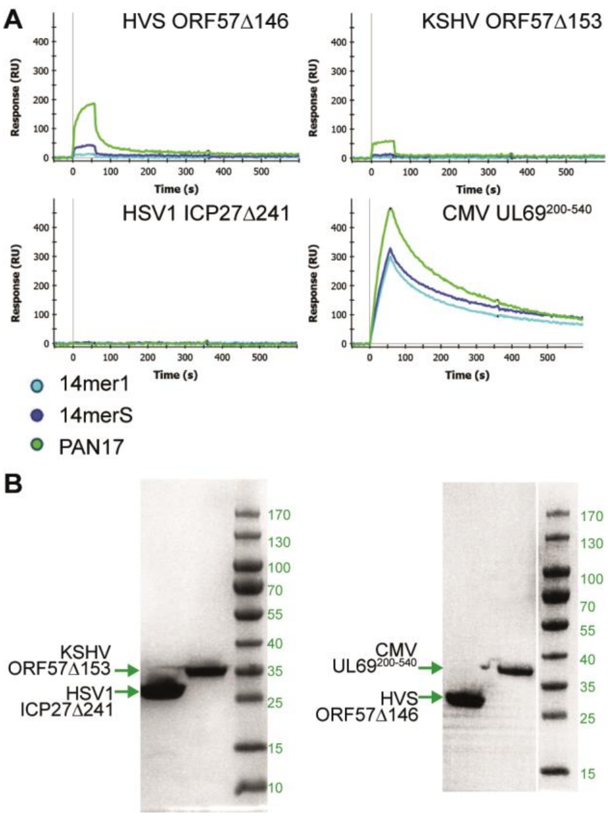

Figure S2. Surface plasmon resonance sensorgrams from RNA binding screen. (A) Each graph represents the response measured from a different protein construct vs each of the three immobilized RNA oligonucleotides 14mer1 (CCGUCCCCGCCGCU), 14merS (CAGUCGCGAAGAGG) and PAN17 (CACCUAUGGAUUUUGUG), colored cyan, blue and green respectively. (B) Coomassie stained SDS-PAGE of purified proteins used in SPR.

```
ORF57      ----------------------------------------------------GPLLKLFD     152
UL69       GPQQQQMLALIDDELDAMDEDELQQLSRLIEKKKRARLQRGAASSGTSPSSTSPVYDLQR     60
                                                                *: .*

ORF57      -----ISILPKSGEPKL-------FLPVPSLPCQEAEKTNDKYVLAMAQRA-MHDVPISS     199
UL69       YTAESLRLAPYPADLKVPTAFPQDHQPRGRILLSHDELMHTDYLLHIRQQFDWLEEPLLR     120
                : : *  .: *:       . *   :  .. *  : .*:* : *:    : *:

ORF57      KQLTANLLPV----KFKPLLSIVRYTPNYYYWV------------------SMRKETIAS     237
UL69       KLVVEKIFAVYNAPNLHTLLAIIDETLSYMKYHHLHGLPVNPHDPYLETVGGMRQLLFNK     180
           * :. ::: *    ::: **:*:  * .*  :                   .**:  : .

ORF57      ANLCTVAAFLDESLCWGQQYLKNDFIFSENGKDIILDTSSALLSQLVHKIKMLPFCHCLM     297
UL69       LNNLDLGCILDHQDGWGDHCSTLKRLVKKPGQMS------A----------WLRDDVCDL     224
            *   :..:**..  **::  . . :..: *:        *           *    * :

ORF57      QTTPQDHIVKQVCYLIASNNRILDAVRYLQTSVIKSPIVLLLAYAVCLPAAI-------I     350
UL69       QKRPPETFS--------------------------QPMHRAMAYVCSFSRVAVSLRRRAL     258
           *. * : :                           .*:   :**. .:  .        :

ORF57      CTKNETQLYSH--CMRILKEYRPGDVMNILHESLTQHLNKCPSSTCAYTTRAIVGTKANT     408
UL69       QVTGTPQFFDQFDTNNAMGTYRCGAVSDLILGALQC--HECQNEMCELRIQRALAPYRF-     315
            ...  *::.:    . :  ** * * :::  :*    ::* .. *    :  :.

ORF57      TGLFFLPTQ------------------     417
UL69       -MIAYCPFDEQSLLDLTVFAGTTTTTA     341
             : : * :
```

Figure S3. Alignment of the primary sequences of IHD regions from HVS ORF57Δ146 and CMV UL69 residues 200-540 reveals 17% identity with several large gaps. The U-bend binding residues conserved in γ-herpesviruses are colored green.

References

1. Sztuba-Solinska, J., Rausch, J.W., Smith, R., Miller, J.T., Whitby, D. and Le Grice, S.F.J. (2017) Kaposi's sarcoma-associated herpesvirus polyadenylated nuclear RNA: a structural scaffold for nuclear, cytoplasmic and viral proteins. *Nucleic Acids Res*, **45**, 6805-6821.
